# CrfP, a fratricide protein, contributes to natural transformation in *Streptococcussuis*

**DOI:** 10.1186/s13567-021-00917-x

**Published:** 2021-03-24

**Authors:** Yinchu Zhu, Jiale Ma, Yue Zhang, Xiaojun Zhong, Qiankun Bai, Wenyang Dong, Zihao Pan, Guangjin Liu, Cun Zhang, Huochun Yao

**Affiliations:** 1 Institute of Animal Husbandry and Veterinary Sciences, Zhejiang Academy of Agricultural Sciences, Hangzhou, 310021 China; 2 College of Veterinary Medicine, Nanjing Agricultural University, Nanjing, 210095 China; 3 Key Lab of Animal Bacteriology, Ministry of Agriculture, Nanjing, 210095 China; 4 OIE Reference Lab for Swine Streptococcosis, Nanjing, 210095 China; 5 College of Animal Science and Veterinary Medicine, Henan Agricultural University, Zhengzhou, 450000 China

**Keywords:** *S.suis*, CrfP, Murein hydrolase, Competence, Virulence

## Abstract

*Streptococcus* *suis* (*S.* *suis*) is an important zoonotic pathogen that causes septicaemia, meningitis and streptococcal toxic shock-like syndrome in its host, and recent studies have shown that *S.* *suis* could be competent for natural genetic transformation. Transformation is an important mechanism for the horizontal transfer of DNA, but some elements that affect the transformation process need to be further explored. Upon entering the competent state, *Streptococcus* species stimulate the transcription of competence-related genes that are responsible for exogenous DNA binding, uptake and processing. In this study, we performed conserved promoter motif and qRT-PCR analyses and identified CrfP as a novel murein hydrolase that is widespread in *S.* *suis* and stimulated with a peptide pheromone in the competent state through a process controlled by ComX. A bioinformatics analysis revealed that CrfP consists of a CHAP hydrolase domain and two bacterial Src homology 3-binding (SH3b) domains. Further characterization showed that CrfP could be exported to extracellular bacterial cells and lytic *S.* *suis* strains of different serotypes, and this finding was verified by TEM and a turbidity assay. To investigate the potential effect of CrfP in vivo, a gene-deletion mutant (Δ*crfP*) was constructed. Instead of stopping the natural transformation process, the inactivation of CrfP clearly reduced the effective transformation rate. Overall, these findings provide evidence showing that CrfP is important for *S.* *suis* serovar 2 competence.

**Supplementary Information:**

The online version contains supplementary material available at 10.1186/s13567-021-00917-x.

## Introduction

*Streptococcus* *suis* (*S.* *suis*) is a gram-positive pathogen responsible for severe economic losses in the global swine industry [1]. In addition, *S.* *suis* is an important zoonotic bacterium that has caused two outbreaks among humans in China [2]. *S.* *suis* can cause septicaemia, arthritis and meningitis in both swine and humans, and over 1600 cases associated with human infection have been reported worldwide. Based on capsular polysaccharides, 33 reference cps loci (*cps* 1–31 and 33 and *cps* 1/2) and 27 novel cps loci (including NCL1-26 and Chz) have been identified for *S.* *suis* strains (with different serotypes and genotypes) to date [3–5]. Moreover, horizontal gene transfer mechanisms have resulted in the diversity of the complex genomes of *S.* *suis* by promoting bacterial evolution through the acquisition of virulence factors and/or antibiotic resistance genes [6].

*Streptococcus* species with the ability to take up naked DNA from the environment and incorporate it into their genomes by homologous recombination are considered competent for natural genetic transformation [7, 8]. This ability has resulted in a variety of genomes and adaptations to diverse environmental conditions. Gene exchange by natural transformation gives streptococci access to a large gene pool that is shared with other closely related commensal streptococci [9]. Consequently, some genes present in the common gene pool confer a selective advantage under certain conditions of stress, such as antibiotic stress, resulting in a rapid spread of these functional genes among the bacteria. In *Streptococcus* *pneumoniae*, the murein hydrolases CbpD, LytA and LytC function as weapons that lyse and kill noncompetent cells and related streptococci via a process called fratricide [10]. In particular, CbpD is controlled by the mechanism of natural transformation. CbpD mainly consists of an N-terminal CHAP (cysteine, histidine-dependent aminohydrolase/peptidase) domain followed by two bacterial SH3 (Src homology 3-binding, SH3b) domains [11]. The CHAP domain is homologous to the corresponding domain of a number of cell wall hydrolases and functions as either an amidase that disrupts the N-acetylmuramyl-L Ala bond or an endopeptidase that cleaves peptide bonds within the stem peptides of bacterial peptidoglycan [12, 13], whereas some evidence suggests that SH3 domains in bacteria are available for cell wall recognition and binding [11]. Because the DNA released from lysed cells can be taken up by competent attacker cells, the rate of gene transfer is greatly increased.

Although many streptococcal species possess genes encoding CbpD-like proteins, homologues of *cbpD* genes have not been found in *S.* *suis* and other streptococci, such as *S.* *gordonii*, *S.* *sanguinis*, and *S.* *mutans* [11, 14]. The fratricide mechanism is regulated by natural transformation, which suggests the presence of a functional interaction between these two systems. We thus questioned whether *S.* *suis* harbours the *cbpD*-like gene that evolved to facilitate the acquisition of homologous donor DNA and contributes to natural transformation. In *S.* *suis*, natural transformation is regulated by the ComRS system, which differs from the two-component signalling systems (TCSs) in *S.* *pneumoniae* [15]. When *S.* *suis* grows under conditions that allow competence, it secretes a peptide pheromone that binds to the ComR regulator and activates the alternative factor ComX. This alternative sigma factor ComX interacts with the RNA polymerase to initiate the transcription of transformasomes by binding to a conserved DNA motif. As a result, ComX-binding motifs are always present in the promoter regions of these genes.

In the current study, we searched the genomes of *S.* *suis* for possible *cbpD*-like genes or substitutes based on the characteristics of CbpD. A novel murein hydrolase termed CrfP was identified in *S.* *suis* strains of different serotypes, the transcription of CrfP is upregulated under XIP stimulation, and this protein serves as a homologue of CbpD. We investigated the roles and relative contributions of CrfP to fratricide in planktonic cultures of *S.* *suis*. Cellular localization studies demonstrated that the SH3b domains of CrfP bind specifically to peptidoglycan, and the CHAP domains act as endopeptidases or amidases. These findings provide valuable insights into the natural transformation.

## Materials and methods

### Bacterial strains, plasmids, and culture conditions

The virulent *S.* *suis* serovar 2 strain ZY05719 (belonging to ST7, as determined through a MLST analysis; GenBank accession NZ_CP007497.1) was isolated from pigs that died from acute septicaemia in Sichuan, China [16]. The strain was cultivated in Todd–Hewitt broth (THB; Becton–Dickinson, New Jersey, USA) at 37 °C in an atmosphere with 5% CO_2_ and plated on Todd–Hewitt agar (THA) with 5% sheep blood. The cells were harvested at the mid-exponential growth phase (OD_600_ of approximately 0.6). *Escherichia* *coli* MC1061, DH5α and BL21 (DE3) cells were cultured in Luria–Bertani (LB) medium (Becton Dickinson, New Jersey, USA) at 37 °C. If needed, various antibiotics were added to the medium at the following concentrations: 50 μg/mL spectinomycin (Spc^R^) and 50 μg/mL kanamycin (Kan^R^) for *E.* *coli* and 100 μg/mL spectinomycin for *S.* *suis*. The *Streptococcus* plasmid pSET-2::*spa* was used in this experiment. This plasmid was extracted using the Takara Plasmid Purification Kit (TaKaRa, Dalian, China). Total genomic DNA was extracted using an Omega Bacterial DNA Kit (Omega, Beijing, China) according to the manufacturer’s instructions. The secondary antibodies were purchased from Beyotime, Shanghai, China.

All bacterial strains and plasmids used in this study are listed in Additional file 1.

### Bioinformatics identification of the candidate hydrolase

Protein sequences of *Streptococcus* ComX-controlled murein hydrolases from different species were retrieved from the National Center for Biotechnology Information (NCBI) database, and the corresponding gene products have the locus tags listed in Additional file 2. Functional predictions of these genes were performed using HHpred and Phyre2 [17, 18]. The BLAST program (available from the NCBI website) was used to identify homologs of fratricide-related proteins in *S.* *suis* based on their highly conserved N-terminal CHAP domains, and candidate target genes were screened by qRT-PCR. Phylogenetic analyses were performed following the procedures outlined by Bingle et al. [19]. A ClustalW alignment was performed using the amino acid sequences of CbpD-like proteins from different *Streptococcus* spp. Thus, a phylogenetic tree of CbpD-like proteins in various *Streptococcus* strains was performed using MEGA (v5.0.3), the neighbour-joining method with Poisson correction and 1000 bootstrap replicates. In addition, the target protein CrfP in different serotype strains of *S.* *suis* was subjected to comparative analysis with DNAMAN. The three-dimensional structures of CrfP were predicted using the SWISS-MODEL online server [20, 21].

### Determination of the transcription levels of the candidate hydrolase

Competence was induced as previously described [22, 23]. Briefly, *S.* *suis* ZY05719, Δ*comR* and Δ*comX* were grown until an OD_600_ of 0.045 was reached. Ten millilitres of culture was collected, and donor DNA (pSET-2, 2 µg) was added to the bacteria along with synthetic XIP (GNWGTWVEE) at a final concentration of 250 µM. The induced cultures were collected 20 min after the addition of XIP. Ten millilitres of uninduced cultures were collected at the same time. The samples were centrifuged for 2 min at 12 000 × *g*, and RNA from the *S.* *suis* strains was extracted using TRIzol (TaKaRa) according to the manufacturer’s instructions.

After extraction as described above, total RNA was reverse transcribed using the HiScript II First-Strand cDNA Synthesis Kit (Vazyme) following the recommended protocol. The QuantStudio 6 Flex Real-Time PCR System (Thermo Fisher Scientific) and ChamQ™ Universal SYBR qPCR Master Mix (Vazyme) were used according to the manufacturers’ instructions. qRT-PCR was performed to compare the RNA transcription levels between the induced and uninduced bacterial cultures. The sequences of the primers used for qRT-PCR are shown in Additional file 3, and the housekeeping gene *parC* was used as a control [24]. The procedure was repeated three times for each sample. The relative fold changes in expression were calculated using the 2^−ΔΔCT^ method [24].

### Expression and purification of the candidate hydrolase

Routine DNA manipulation, including mainly amplification and ligation, was performed as previously described [25]. The restriction and ligation enzymes were purchased from Takara. DNA sequencing was performed by GenScript Biotechnology Co., Ltd.

We constructed the main CrfP, CHAP domain and GFP-SH3b fusion protein with the pET-28a vector. The sequences for *crfP* and the two domains were amplified using ZY05719 DNA as a template. The gene encoding GFP was then amplified by PCR from pKSM410.

The SH3b and GFP PCR products were fused together by overlap-extension PCR. The expression vector pET-28a was digested with *NcoI* and *XhoI* and ligated with the PCR fragments by homologous recombination. The resulting recombinant vector was submitted for sequencing. The other shuttle vector pSET-2-*crfP*-*spa* was constructed using the same method.

### Knockout of the hydrolase gene *crfP*

To investigate the contribution of the *crfP* gene in *S.* *suis*, Δ*crfP* was obtained using the novel natural DNA transformation method [23, 26]. Here, this mutant strain was constructed by two steps of natural transformation. First, the up/downstream homologous fragments were amplified by PCR with the primers crfP1/crfP2 and crfP3/crfP4, respectively. The *sacB-spc* cassette was used for resistance selection, and sucrose was used for negative selection. The DNA products were mixed with the peptide, and bacteria that grew on Spc THB-agar medium were used as the target gene-knockout strain. Second, the homologous fusion fragment, without any marker or amplification, was re-transferred to the protopositive mutant. The Δ*crfP* mutant was screened on THB-agar medium containing 10% (w/v) sucrose. The other mutant strains, namely, Δ*comR* and Δ*comX*, were constructed using the same procedure.

All primers used in this study are listed in Additional file 3.

### Western blotting

Strain ZY05719 with pSET-2-*crfP*-*spa* was grown to the mid-log phase, and the cultures were then centrifuged at 4 °C. The supernatant and bacterial pellet were collected. The supernatant was filtered (0.22 μm) to remove residual bacteria. Trichloroacetic acid was added to the filtrate to a final concentration of 15%, and the solution was incubated on ice for approximately 30 min. After centrifugation, the pellet was washed twice with prechilled acetone and then air dried.

The bacterial pellet was washed twice with PBS and resuspended in 1× SDS-PAGE sample loading buffer (as the whole-cell proteins). The secreted proteins and whole-cell proteins from the above-mentioned samples were used for Western blot analysis. The proteins were separated by SDS-PAGE, transferred to nitrocellulose membranes, probed with antibodies against FLAG, and detected by HRP-conjugated IgG anti-rabbit antibody (Beyotime). GroEL was used as a loading control. The Tanon™ High-sig ECL Western blotting Kit (Tanon) was used for signal detection. The image was obtained with a Tanon 5100 apparatus (Tanon).

### TEM and immunofluorescence microscopy

The colony morphology was analysed by TEM. Briefly, bacteria growing to the mid-exponential phase were resuspended in PBS, and the suspension was incubated with CrfP for 15 min and 30 min. The specimens were then harvested by centrifugation and fixed in 2.5% glutaraldehyde for more than 24 h. The samples were dehydrated in propylene oxide for 10 min, embedded in epoxy resin, and examined using a Hitachi H-7650 system (Hitachi) according to the manufacturer’s instructions.

CrfP-GFP or SH3b-GFP was incubated with *S.* *suis* for 60 min, and the pellet from 1 mL of culture was washed once in PBS. Five microlitres of this suspension was transferred to a glass slide for drying, and 4′,6-diamidino-2-phenylindole (DAPI) was used to stain the cells. After a coverslip was positioned over the sample, the slide was visualized and imaged with a laser-scanning confocal microscope (Nikon Instruments, Inc., Leica Sp5 AOBS confocal system).

### Determination of the lytic spectrum of the candidate hydrolase

The lytic specificity of CrfP was detected by a turbidity assay [27, 28]. Selected bacterial strains (Additional file 1) were grown to an OD_600_ of 0.8, washed with PBS and adjusted to an OD_600_ of 1. Subsequently, 20 µg of protein was added in each test, and PBS was used as a negative control. Each culture was incubated at 37 °C for 15 min, and the turbidity at OD_600_ was measured. The percent reduction in turbidity was calculated according to the readings.

### Natural transformation test

The natural transformation protocols for *S.* *suis* were described in detail in a previous report. The main process was as follows. *S.* *suis* strains were grown in THB broth at 37 °C in an atmosphere with 5% CO_2_ until the OD at 600 nm reached approximately 0.04–0.06. Subsequently, 100 μL of the *S.* *suis* culture was mixed with 5 μL of peptide pheromone along with the DNA products (the shuttle vector pSET-2 was used as the template in the test). After 2 h of incubation, the samples were diluted tenfold and plated on THB agar plates with spectinomycin. Different selection conditions were prepared. The positive colonies were then observed on the selection plates, and the frequency was calculated to enumerate the colony-forming units (CFUs) [23].

### Growth curves and biofilm assay

To assess the influence of the target genes on the growth rate of *S.* *suis*, the growth kinetics of ZY05719 and mutant strains were determined. Briefly, the bacteria were cultivated in THB medium at 37 °C with shaking until an OD_600_ of approximately 0.6 was reached, the medium was replaced with fresh medium, and the optical density of each culture was monitored at 1-h intervals by spectrophotometry. The same logarithmic-phase cultures of the strains were diluted to an OD_600_ of approximately 0.2, and 200 μL of the suspension solution was inoculated in 96-well polystyrene plates. Human plasma fibrinogen (2.5 mg/mL) was mixed with the bacterial culture prior to their transfer to plates because *S.* *suis* serotype 2 biofilm formation requires the presence of fibrinogen as previously reported [29]. After incubation for 24 h, the plates were washed with PBS to remove planktonic bacteria. Methyl alcohol was added to fix the bacterial cells, and the cells were then washed three times with PBS. Finally, the biofilm was stained with 0.1% crystal violet, and the crystal violet was dissolved with 95% ethyl alcohol. The OD_595_ values were then detected with a microplate reader [30].

### Statistical analysis

The data from all the experiments were plotted using GraphPad Prism (v.5) software for statistical analyses. The data are presented as the mean values ± standard errors of the means (SEMs). Unpaired two-tailed Student’s *t* test and nonparametric Mann–Whitney U test were used. A *P* value of < 0.05 was considered significant.

## Results

### CHAP domain-containing genes are significantly upregulated in response to competence induction

Studies have revealed that the fratricide-related protein CbpD in streptococci is synthesized when the cells enter a competent state, as observed in *S.* *pneumoniae*, *S.* *thermophilus* and *S.* *mitis* [31]. We then questioned whether *S.* *suis* produces the CbpD-like protein or harbours an alternative murein hydrolase that performs a similar function. It is well known that the CHAP domain is an important element for the lytic activity of the CbpD enzyme. We then searched the genome of the *S.* *suis* serotype 2 strain ZY05719 by BLASTP using the CHAP domain and identified four putative proteins, namely, ZY05719_00195, ZY05719_04700, ZY05719_09810 and ZY05719_10070, as potential target fratricide-related proteins.

**Figure 1:**
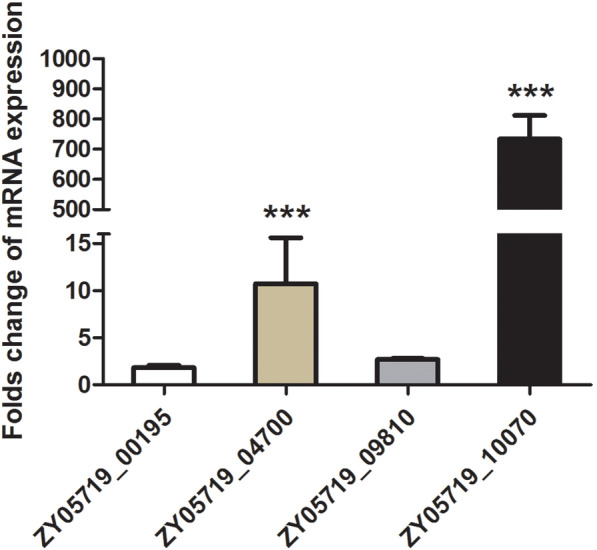
**Differences** **in** **the** **transcription** **of** **CHAP** **domain-containing** **genes** **after** **the** **induction** **of** **competence.** The genes ZY05719_04700 and ZY05719_10700 were significantly enriched by XIP induction. ZY05719_00195 and ZY05719_09810 exhibited no relationship with competence. The data were normalized to the transcription of the housekeeping gene *parC*. The relative expression levels are presented as the means ± SDs from three biological repeats (***P < 0.01).

To determine whether these genes were regulated under peptide pheromone stimulation in the competence system, quantitative real-time PCR was performed. As shown in Figure 1, the expression of the ZY05719_04700 and ZY05719_10070 genes was upregulated by more than tenfold, and that of ZY05719_10070 was upregulated by more than 600-fold, which suggested that both ZY05719_04700 and ZY05719_10070 might be involved in competence. However, ZY05719_00195 and ZY05719_09810 did not respond to the inducing peptide. Additionally, the results showed that ZY05719_04700 is located in a pathogenicity island (PAI) designated 89 K, which is specific for epidemic isolates from Chinese outbreaks but not for other clinical isolates such as P1/7 or CZ130302. Therefore, this result suggests that ZY05719_10070 is a possible candidate hydrolase related to competence, and this protein was thus selected for further study.

### Bioinformatics analysis of ZY05719_10070 as a potential hydrolase related to fratricide in *S. suis*

**Figure 2:**
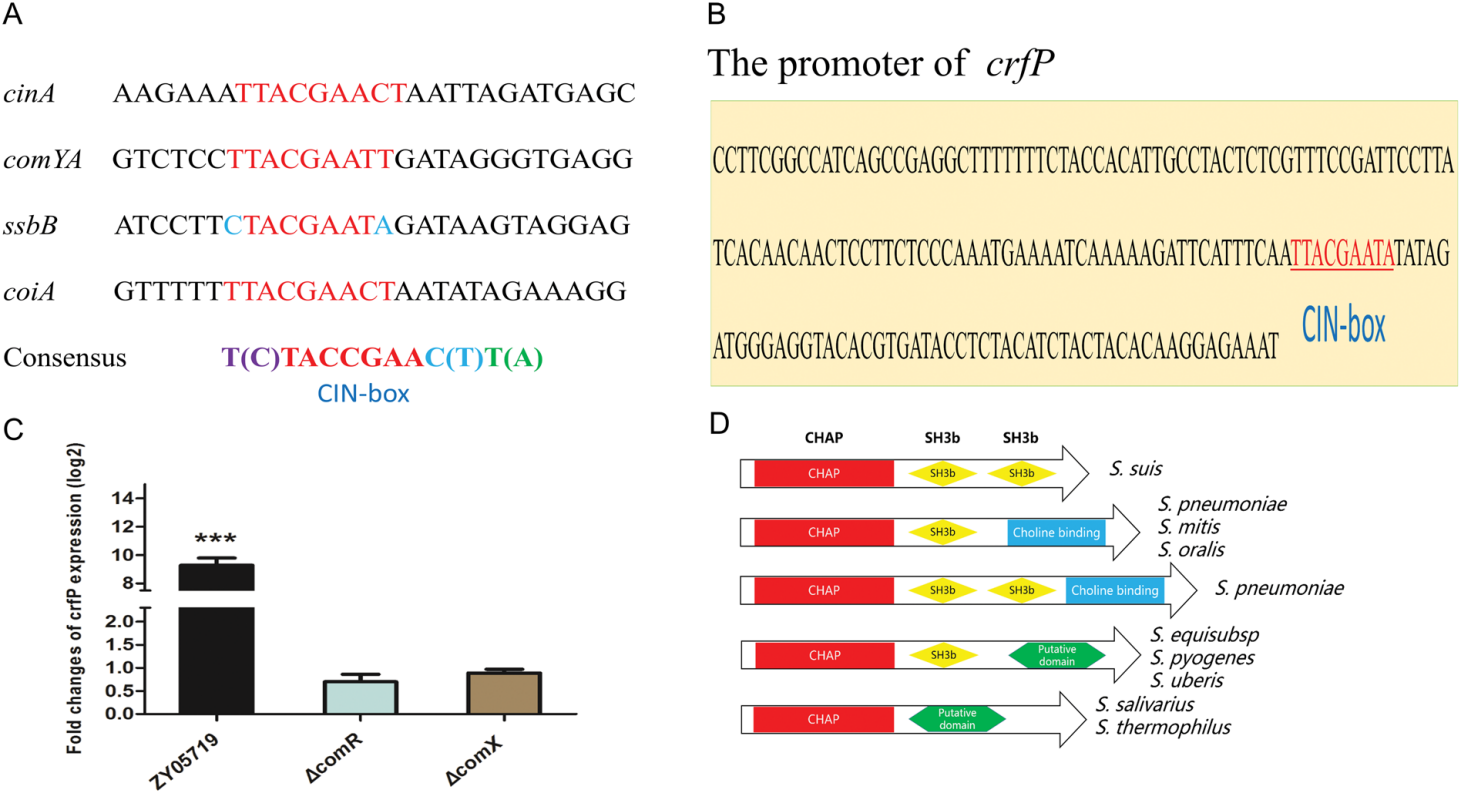
**Structural** **analysis** **of** **CrfP** **homologous** **proteins** **in** ***Streptococcus.*** **A** Promoter motif (CIN box) of natural transformation-related core genes regulated by ComX. **B** Conserved motif in the *crfP* gene promoter. **C** A qRT-PCR analysis showed that *crfP* was influenced by ComR and ComX under competence. The transcription level did not increase in the absence of the *comR* or *comX* gene. **D** Domain organization of murein hydrolases from different species of streptococci: CHAP, cysteine, histidine-dependent amidohydrolases; SH3, binds peptidoglycan; choline-binding, binds choline residues linked to teichoic acid; conserved domain, uncharacterized domain. The gene from each streptococcal species has the locus tags listed in Additional file 2.

Previous studies on competence in *Streptococcus* species have demonstrated that the most essential structural elements are under the control of the sigma X factor, that is, the regulator ComX [8, 32]. In *S.* *suis*, core genes are regulated by ComX, such as *cinA*, *recA*, *comYA*, *comYB*, *ssbB*, and *cinA*, via the induction of competence, as determined by microarray transcriptome analysis [15]. A MEME analysis showed that these genes harbour the CIN-box motif YTACGAAYW in the promoter region. Interestingly, the gene ZY05719_10070 encoding a putative murein hydrolase had a special motif, TTACGAATA, in the promoter region, which suggested that it was a target gene of the ComX regulon (Figures 2A, B). To determine whether the conserved protein was an important element for competence, we generated several deletion mutants of the *comX* or *comR* gene and verified the deletions by sequencing. The qRT-PCR results showed that the ZY05719_10070 gene was not upregulated by small-peptide induction without ComX or ComR (Figure 2C). As demonstrated by sequence analysis, ZY05719_10070 encodes a 30-kD protein consisting of an N-terminal CHAP domain and two C-terminal SH3b domains. Indeed, a signal peptide sequence in the N terminus of this protein was predicted using SignalP server 5.0 software (Additional file 4A). The signal peptide usually suggests the regulation of the transmembrane secretion of extracellular proteins, which indicate the protein encoded by ZY05719_10070 could be secreted into environment. The CHAP domain is widely found in the Trypanosomidae family of bacteria, archaea and phages and has been proposed to function as a peptidoglycan hydrolase [13]. By screening proteins that were highly homologous to CbpD or CbpD-like proteins, we found that the integration of this structure was similar or equal to that of the homologues in *S.* *pneumoniae*, *S.* *oralis*, and *S.* *equi* subsp. In addition, a choline-binding domain was found in the C terminus of the *S.* *pneumoniae* protein*.* Although the choline-binding domain was absent in the proteins in *S.* *equi* subsp*.*, *S.* *pyogenes* and *S.* *suis*, the *S.* *oralis* protein harboured an unknown domain. In *S.* *suis*, the protein encoded by the gene ZY05719_10070 was redesignated CrfP (ComX-related fratricide protein, CrfP) due to the absence of a choline-binding domain (Figure 2D).

Moreover, a phylogenetic analysis based on amino acids distributed these proteins into three branches and showed that these homologous proteins from diverse species likely originated from the same ancestor. The homologues from *S.* *suis* strains with different serotypes belong to the same group, and those from *S.* *pneumoniae*, *S.* *oralis* and *S.* *mitis* constitute the second group, whereas the proteins in *S.* *equi.* subsp*.*, *S.* *pyogenes*, *S.* *salivarius* and *S.* *thermophilus* are far from the two above-mentioned groups, which is consistent with the results from the domain structure analysis. The results also showed that the homologues from *S.* *suis* are strongly conserved, regardless of the complex serotypes or genotypes, such as the novel serotype strains CZ130302 and AH681 (Additional file 5).

### Complex domains of CrfP responsible for different roles in the lytic process

To determine the specific roles of CrfP in vitro, the main protein was expressed in host *E.* *coli* BL21 with the pET-28a vector as a C-terminally His6-tagged protein. The protein was purified by Ni+ affinity chromatography and then identified by Coomassie blue staining and Western blotting with a monoclonal His tag antibody, which showed a recombinant protein with a molecular mass that was consistent with the predicted size of approximately 35 kD (Additional file 5).

**Figure 3:**
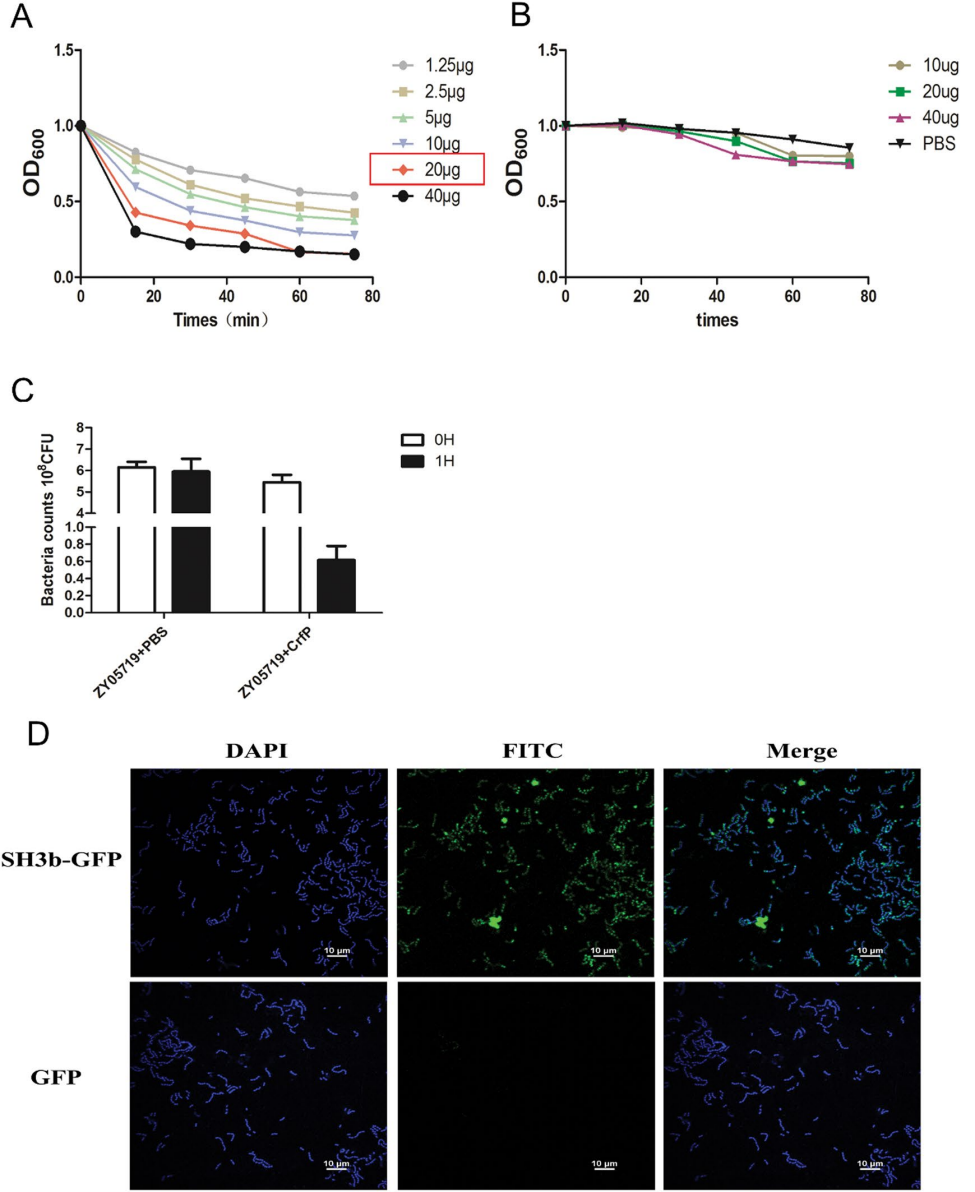
**Analysis** **of** **the** **role** **of** **CrfP** **in** **the** **lytic** **process.** **A** Decreases in the turbidity of *S.* *suis* serovar 2 ZY05719 treated with different doses of CrfP were observed at five time points: 15 min, 30 min, 45 min, 60 min and 75 min. CrfP was used, and the turbidity showed a sharp decrease within 15 min. After incubation for 60 min, the lytic activity of 20 μg/mL CrfP was very close to that of 40 μg/mL CrfP; therefore, we selected 20 μg/mL as the optimal final concentration of CrfP. **B** Only the CHAP domain-containing protein was added to bacteria, but no lytic ability was observed within the incubation period. **C** ZY05719 cells were treated with CrfP (20 μg) for 1 h, and the live bacterial counts were determined. **D** Binding of purified exogenous SH3b–GFP fusion protein to the surface of *S.* *suis* cells. Fluorescence micrograph showing *S.* *suis* cells stained with the DNA-specific fluorescent probe DAPI (blue) and the fusion protein (green). GFP protein was used as the control. The white bars represent 10 μm.

**Figure 4:**
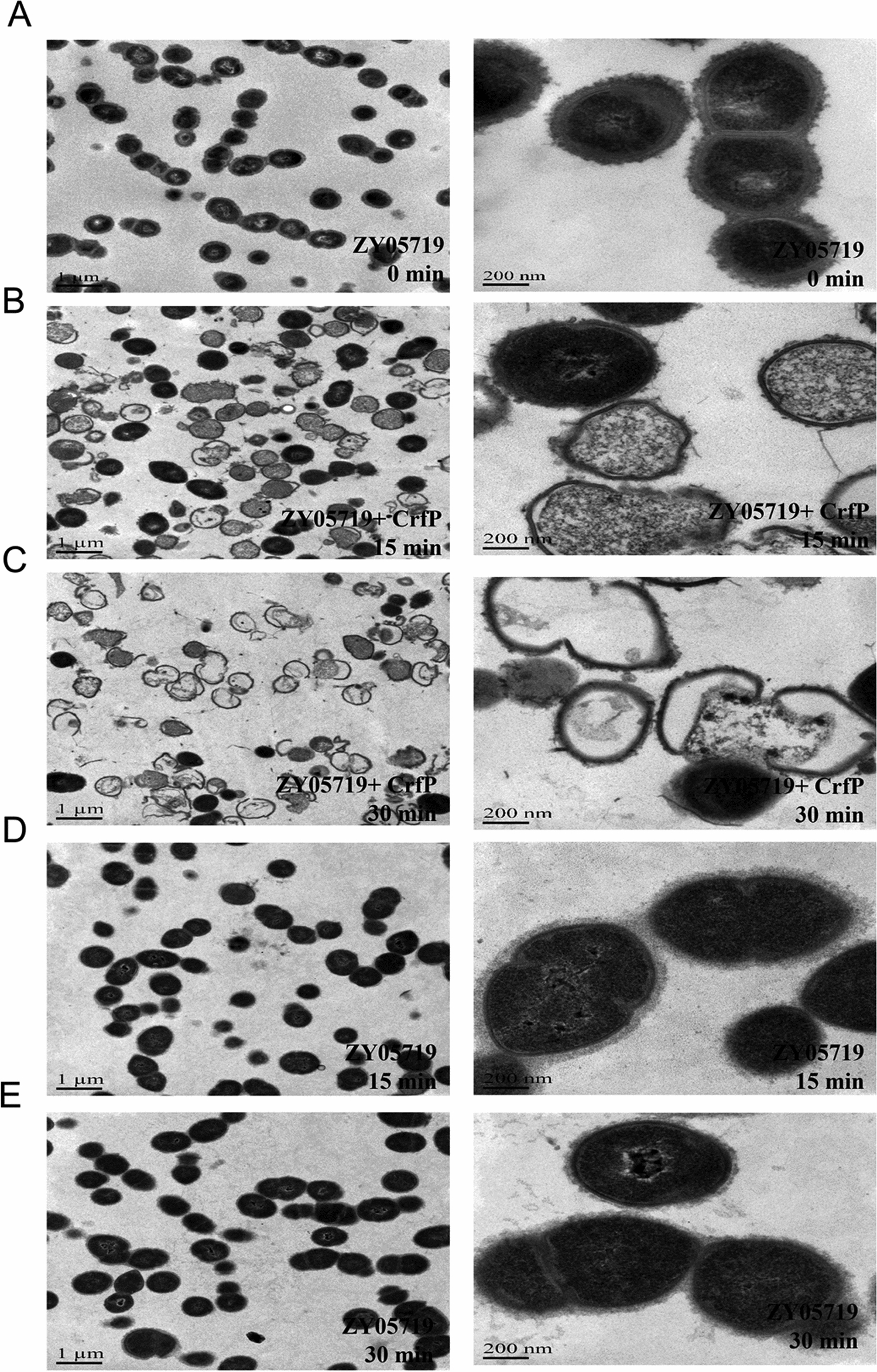
**TEM** **assay** **of** **CrfP** **lytic** ***S.*** ***suis*** **serovar** **2** **ZY05719** **cells.** **A** ZY05719 diluted with PBS at the beginning of the experiment. **B** ZY05719 incubated with CrfP for 15 min. The cytoplasm of ZY05719 spilled over from the hole. **C** Cell wall degradation after incubation with CrfP for 30 min. The extrusion and loss of cytoplasmic contents were observed, and these effects only a cell wall “ghost”. **D**, **E** ZY05719 with no CrfP served as a negative control.

Furthermore, we explored the hydrolytic activity of CrfP through a turbidity assay. The results showed that the decrease in the turbidity of *S.* *suis* ZY05719 cells was obvious after coincubation with different concentrations of CrfP. The lytic activity of 20 μg/mL CrfP was similar to that of 40 μg/mL CrfP; therefore, we selected 20 μg/mL as the working concentration of CrfP for the subsequent experiments. In general, the turbidity decreased sharply within the first 15 min (Figure 3A). As expected, after 1 h of coincubation with CrfP, the OD_600_ of *S.* *suis* decreased from 1 to approximately 0.1–0.2, and the live bacterial cell count decreased more than 90% (Figure 3C). Furthermore, the changes in the shape of the bacterial cells were investigated by TEM. As depicted in Figure 4, the structure of *S.* *suis* ZY05719 retained a short-chain form in PBS without CrfP at 15 min and 30 min, whereas rupture of the cell wall due to the lytic activity of CrfP in PBS resulted in partial or total loss of the cytoplasmic contents and formation of a cell ghost. CHAP and SH3b proteins were produced to determine the individual effects of each domain. However, the same turbidity assay showed that the CHAP domain alone could not lyse *S.* *suis* cells even at higher concentrations (Figure 3B). Based on the available evidence regarding the recognition of peptidoglycan by bacterial SH3b, it is reasonable to assume that the CHAP domain plays a lytic role through assistance by the SH3b domain to capture target cells. Thus, we verified the potential correlation between SH3b and the bacterial cell wall. The SH3b recombinant protein fused with a GFP marker at the C terminus was simply purified with a His tag. Fluorescence micrographs revealed green fluorescence around *S.* *suis* cells after coincubation with the recombinant protein, which confirmed that this fusion protein could bind directly to the cell surface. As a negative control, GFP did not bind to *S.* *suis* (Figure 3D). Altogether, these results demonstrate that the CHAP domain could induce the lysis of target cells and that this effect is accompanied by the binding of the SH3b domain to the cell wall surface of *S.* *suis*.

### Subcellular localization of *CrfP*

**Figure 5:**
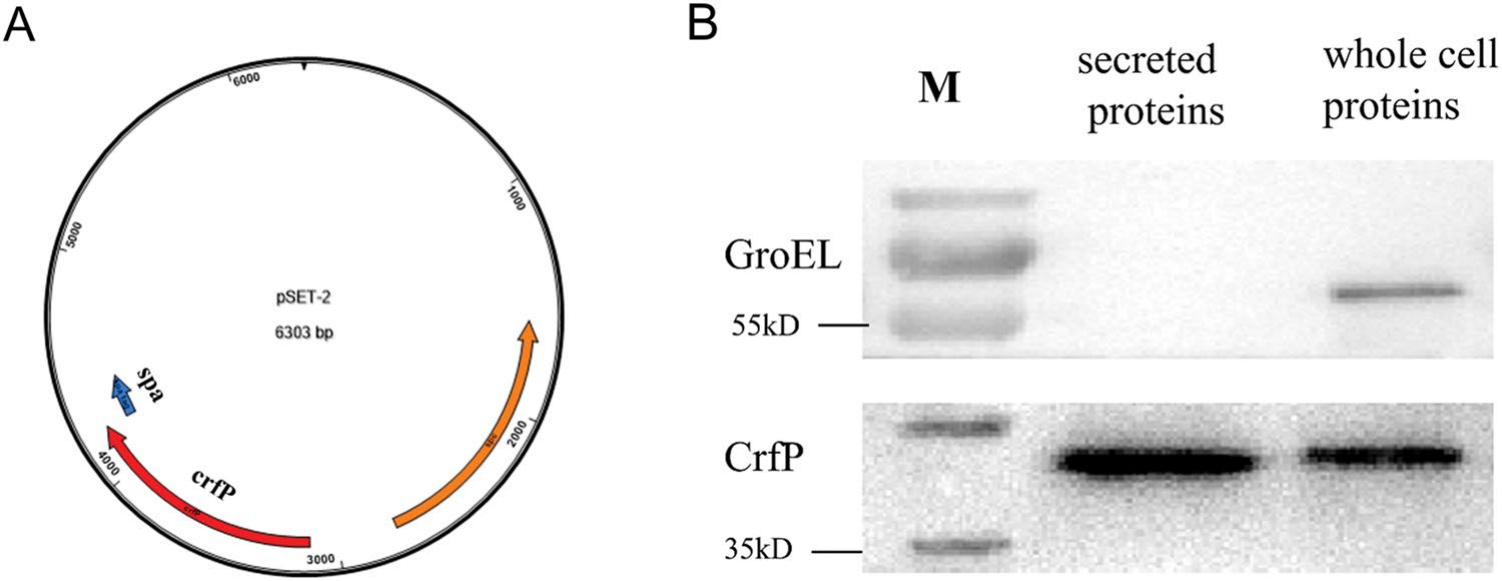
**CrfP** **could** **be** **secreted** **from** **the** **cell.** **A** Construction of the vector pSET-2-*crfP*-*spa.* **B** Western blot analysis of CrfP. Total cell proteins and secreted proteins extracted from strain ZY05719 with pSET-2-*crfP*-*spa* were probed with anti-FLAG serum and anti-GroEL serum. Only CrfP was present in the secreted proteins, but both GroEL and CrfP were present in the total cell proteins.

The present results revealed that CrfP could attach to and attack the *S.* *suis* cell wall. It was thus reasonable to speculate that this protein should be secreted outside the bacterial cell to facilitate contact with the cell surface. The *crfP* gene was cloned into a shuttle vector fused with an SPA tag (3× flag) and transferred into ZY05719 to overexpress the CrfP protein. A Western blot analysis was performed to investigate whether CrfP would be secreted into the bacterial culture supernatant, and this analysis was performed with a FLAG-tagged antibody against SPA. Notably, our data showed that CrfP was captured from both the secreted proteins and the total *S.* *suis* proteins and was detected as a predicted band of approximately 35 kD. As a control, the cytoplasmic protein GroEL could not be detected in the supernatant sample but was present in the total cellular proteins (Figure 5). This observation confirmed that CrfP is a component of the secreted proteins.

### *CrfP* displayed a narrow lytic spectrum for only *S. suis* in vitro

**Figure 6:**
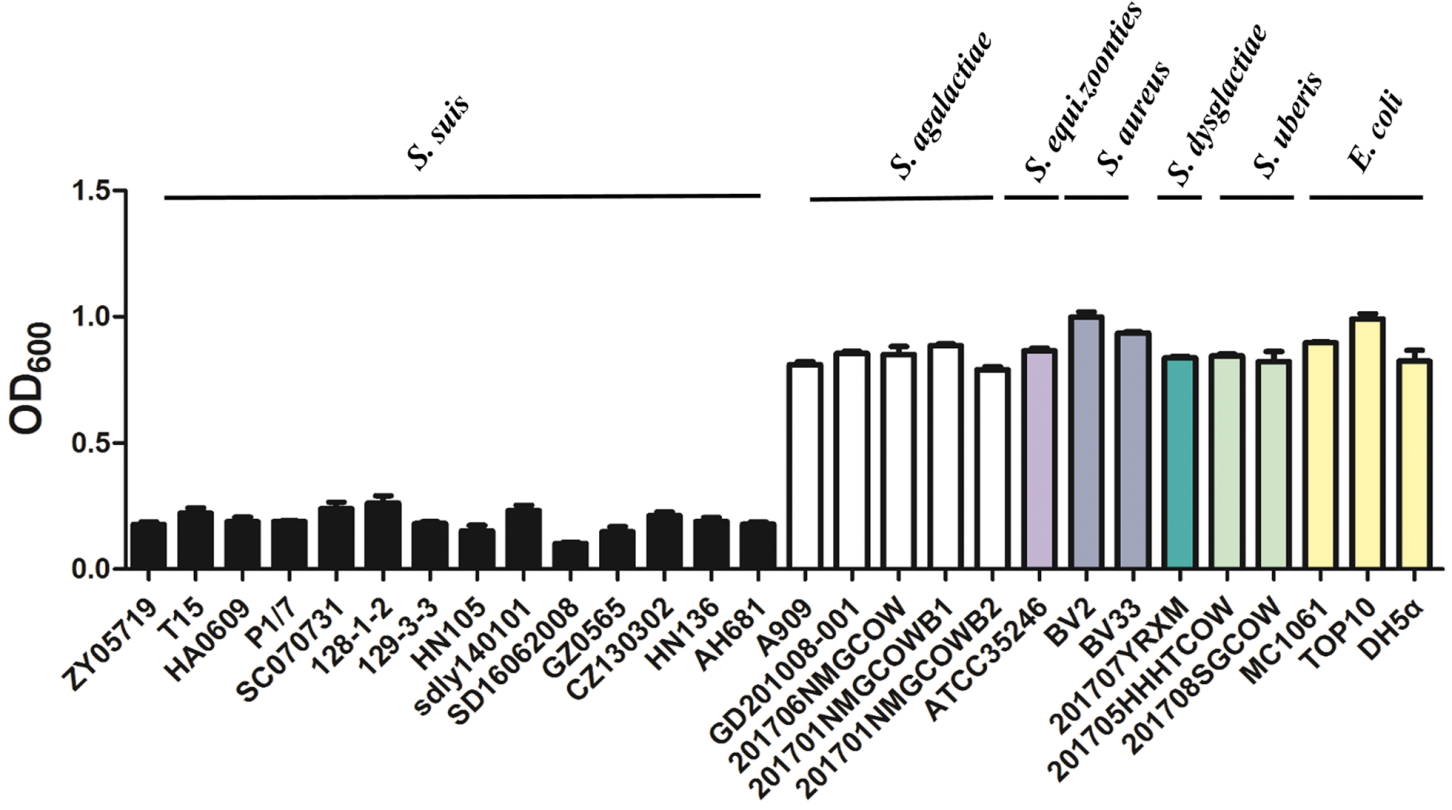
**Spectrophotometric** **lysis** **assays** **of** **CrfP.** The activity of CrfP was measured by determining the decrease in the OD_600_ within 1 h. CrfP showed strong lytic activity against *S.* *suis*, including different serotypes but exhibited no lytic activity against *E.* *coli* or other *Streptococcus* species.

Although CrfP has been confirmed to be a murein hydrolase in *S.* *suis* serovar 2, whether CrfP could lyse strains of different species due to the widespread distribution of fratricide-related homologous proteins among diverse streptococci remains unclear. To gain insight into the specificity of CrfP, lytic spectrum analyses of 28 strains of different species were performed for further verification. The different bacteria were suspended in PBS to a final OD_600_ of 1.0, and the decrease in the OD_600_ within 1 h was determined to estimate the activation of CrfP. The results showed that CrfP could strongly lyse all the tested *S.* *suis* strains (including different serotypes), as demonstrated by a decrease in the turbidity to 0.1–0.2, but the OD_600_ values for other tested species of bacteria, such as *S.* *agalactiae*, *S.* *equi.* *subsp.*, *S.* *aureus* and *E.* *coli*, did not exhibit significant decreases, which suggests that CrfP has a narrow muralytic spectrum (Figure 6).

### Contribution of CrfP during natural transformation

**Figure 7:**
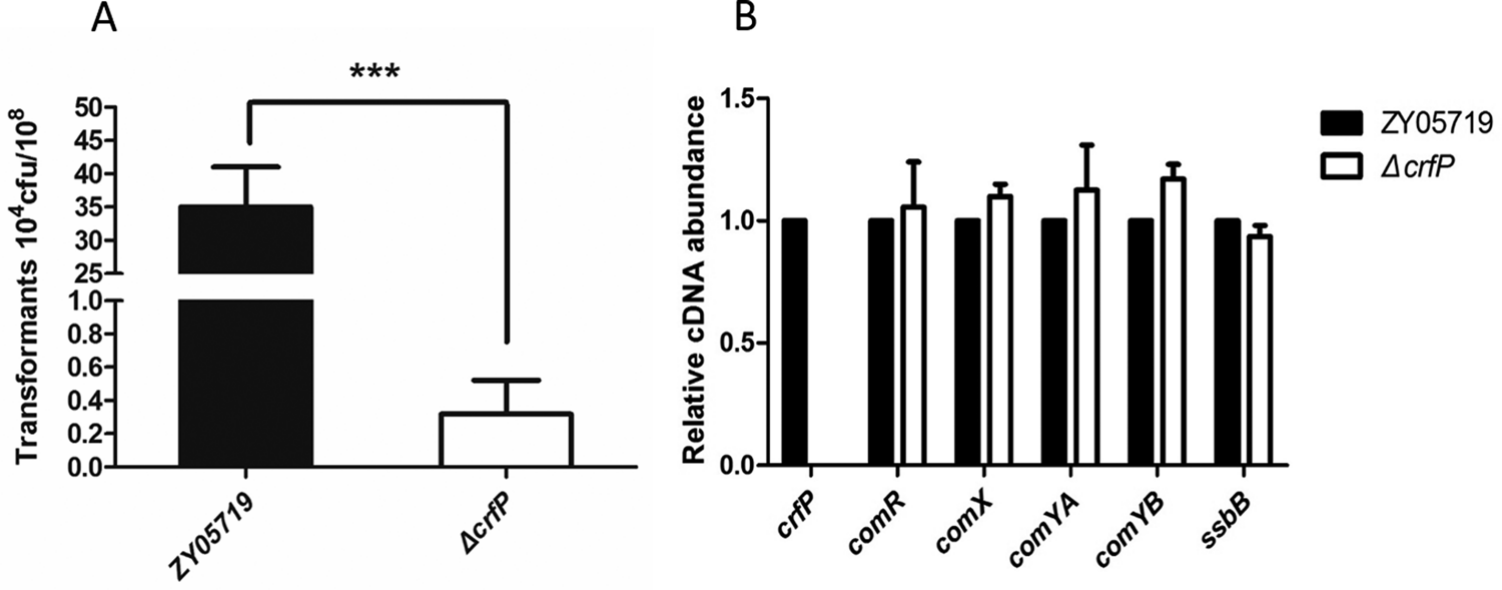
**CrfP** **contributes** **to** **natural** **transformation** **in** ***S.*** ***suis.*** **A** The deletion *of* *crfP* attenuated the transformation ability. **B** The qRT-PCR analysis showed that *crfP* does not play roles in competence-related processes. The transcription level did not change in the absence of the *crfP* gene. Unpaired two-tailed Student’s *t* test was used for the statistical analysis (*P < 0.05).

CrfP is a murein hydrolase regulated by ComX and enhances transcription by inducing peptides that accelerate the transformation process. Some studies have noted that fratricide-related proteins could disrupt the cell wall, which is absolutely required for the liberation of chromosomal DNA from noncompetent cells cocultured with competent cells. Thus, an analysis of the efficiency of natural transformation was performed to verify whether CrfP is needed for the transformation. As demonstrated in Figure 7A, the ability of the Δ*crfP* mutant to obtain pSET-2 vectors was significantly weakened compared with that of the wild type. Few positive clones were present on THB plates with the antibiotic Spc, and deletion of the *crfP* gene resulted in a more than 80-fold decrease in the DNA transformation efficiency. Further qRT-PCR assays showed that the loss of *crfP* did not influence essential genes, such as *comYA*, *comYB*, and *ssbB*, which are known membrane-associated genes, and pilus genes needed for DNA entry, binding and uptake (Figure 7B). These data suggest that CrfP was important but not essential in the transformation process. Taken together, these results have confirmed the important role of CrfP in the competence of *S.* *suis*.

## Discussion

Natural transformation is an important horizontal gene transfer mechanism in streptococcal species. More than 80 types of bacteria have been confirmed to have this ability [33]. Moreover, previous studies have revealed that natural transformation is beneficial to genetic variation and that several elements could contribute to bacterial growth, environmental fitness and pathogenicity. Despite detailed research on *S.* *pneumoniae* and *S.* *mutans*, few studies have focused on competence in *S.* *suis* [34]. Based on the significant differences in the evolution of *S.* *suis* compared with that of other *Streptococcus* species, *S.* *suis* was designated a noncompetent bacterium until 2014 [22]. *S.* *suis* carries the ComRS-ComX regulatory system to control the pheromone-induced mechanism underlying its competence, as obtained with *S.* *mutans* [23]*.* However, the roles of most factors involved in *S.* *suis* competence, such as those of the murein hydrolases reported here, remain unclear.

Pheromone-induced competence results in DNA exchange and recombination in bacteria. Although strains can be fed DNA in the laboratory environment, they must produce exogenous nucleic acids themselves in the natural environment to provide the material for DNA recombination. Therefore, *Streptococcus* species evolved a fratricide mechanism involving the hydrolases CbpD and LytF, the autolysins LytA and LytC, or the bacteriocin production protein CibAB to generate a gene pool [10, 11, 35, 36]. As determined in previous studies, these hydrolases could target the cell wall with the help of the CHAP domain and belong to competence-related proteins containing a CIN box in the promoter region, which could be regulated by ComX. Several previous findings have shown that CbpD preferentially targets and ruptures the division zone, and a possible explanation is that the division zone represents the weakest part of the cell wall [11]. In addition, it has been confirmed that the autolysin AtlAss in the novel serotype *S.* *suis* CZ130302 targets the cell division zone via three repeat WG domains and hydrolyses the peptidoglycan with a conserved N-acetyl-muramidase domain, which results in the short chain length in *Streptococcus* [37]. In this study, we identified and characterized a novel CbpD-like protein in *S.* *suis*, CrfP, which consists of CHAP and SH3b domains. The detailed analysis showed a conserved motif, TTACGAATA, present in the CrfP promoter region that matches the CIN box. In this manner, the results showed that CrfP is controlled by the ComR and ComX regulons, which indicates that CrfP forms part of the transformation components. Indeed, our results revealed that the SH3b domain of CrfP could bind to the surface of the cell wall and recognize the cell wall of *S.* *suis.* Thus, the CHAP domain could lyse target cells with the assistance of SH3b; otherwise, the CHAP domain would not exert an effect by itself.

Moreover, it has been proposed that the biological function of CrfP is to lyse and release DNA from susceptible bacteria, including noncompetent or related *Streptococcus* strains sharing a similar genetic background, to provide homologous transformation-related DNA to competent recipient cells. The narrow target spectrum of CrfP for fratricide in *S.* *suis* is consistent with the above-mentioned hypothesis. In addition to restructuring the mutation, the repair of damaged DNA is another effect of natural transformation in streptococci, which occurs only if the competent streptococci possess a mechanism that enables them to capture DNA from closely related bacteria and that will prevent error due to genetic recombination [38]. Otherwise, the above-mentioned observations strongly confirmed that CrfP could kill *S.* *suis* with diverse *cps* types, which indicates that this protein might be a suitable candidate for the prevention of *S.* *suis* infection. Furthermore, the natural transformation frequency data verified that CrfP supported the progression of transformation but was not an essential protein. We may wonder how hydrolases contribute to enhanced transformability. Some researchers have proposed that these hydrolase proteins, via their lytic activity, could stimulate DNA uptake by making the cell wall of bacteria more permeable to DNA and via pseudopili produced during competence [14, 39, 40]. In *S.* *pneumoniae* and *S.* *mutans*, LytF can compensate for the lack of CbpD to affect transformability. Interestingly, most streptococci encode other fratricide-related CbpD-like proteins, but a CrfP-like protein has not been found in *S.* *suis* to date. To uptake DNA, the formation of pilus DNA traps is needed during competence, and we searched the *S.* *suis* genome based on the knowledge of the *S.* *pneumoniae* pilus. Thus, we identified a *com*YA–YH operon in *S.* *suis*, which is homologous to the genes encoding type 4 pili (T4P) in many other *Streptococcus* species (Additional file 2). As previously demonstrated in *S.* *pneumoniae* and *S.* *mutans*, the promoter of *com*YA–YH operon also contains a CIN-box and is regulated by ComX under competence condition.

Moreover, to avoid committing suicide, competent *Streptococcus* could produce the integral membrane protein ComM, which protects bacteria against the muralytic action of CbpD via an unknown mechanism [10, 31]. However, we did not identify the immune protein CrfP based on the protein homology of ComM from *S.* *pneumoniae*. Transcriptional research has demonstrated that the immune protein ComM is expressed prior to fratricin synthesis. In contrast, using published microarray data from *S.* *suis*, we did not find the existence of potential fratricide-related factors in the genes coregulated with CrfP during the development of competence [15]. Notably, not all hydrolases have corresponding immune proteins. To date, a fratricin-specific immune protein has been identified only for *S.* *pneumoniae*, *S.* *pseudopneumoniae*, *S.* *mitis*, *S.* *oralis*, *S.* *infantis*, and *S.* *peroris*, which all carry the *comM* gene [41]. *S.* *gordonii* has a competence-related murein hydrolase, LytF, but researchers have failed to confirm a competence-induced immunity mechanism [42]. Similarly, no immunity-related gene that protects *S.* *thermophiles* against CbpD has been identified. Another study showed that H_2_O_2_ can efficiently inactivate CbpD to oxidize the active-site cysteine to sulfenic acid, which renders the enzyme non-functional during competence in *S.* *thermophiles* [14]. This protection mechanism that prevents massive self-lysis in a competent population without an immune protein has been identified. In *S.* *suis*, since our results only displayed that *S.* *suis* can be lysed at high CrfP concentrations, we failed to confirm whether *S.* *suis* harbours an immune protein based on current information. Further studies should be performed to explore whether similar or unknown protection mechanisms were employed during the competence.

Most streptococci that are known to develop natural competence contain a hydrolase that is tightly expressed during competence development. This finding suggests that the lytic mechanism must be very important for the biology and evolution of streptococci. Thus, another hypothesis is that while the function of hydrolase is not completely understood, our data indicated the existence of a mechanism that encourages competent cells to acquire DNA material from the outside environment (in this case, plasmids) rather than a killing mechanism that eliminates the surrounding bacteria or itself. The increased permeability might contribute to the uptake of extracellular DNA.

An alternative role has been suggested for fratricide, which connects this process to virulence through the release of virulence factors. The human pathogen *Listeria* *monocytogenes*, which is dependent on the continual remodelling of the cell wall with the hydrolase LytA or MurA, could play a significant role in pathogenicity [43]. In *S.* *pneumoniae*, a main pneumococcal autolysin not only inhibits complement-mediated immunity via a complex process involving impaired complement activation but also increases the binding of complement regulators to direct the degradation of complement C3 [44–46].

In summary, our study screened a novel murein hydrolase through genomic alignment and bioinformatics analysis, and this hydrolase is associated with competence in *S.* *suis*.

## Supplementary Information

**Additional file 1. Characteristics of the bacterial strains and plasmids used in this study.**

**Additional file 2. Locus tags of competence-related murein hydrolases and pili from different** ***Streptococcus*** **species.**

**Additional file 3. Primers used in this study.**

**Additional file 4. Structure and Western blot analysis of His tagged CrfP protein.** (A) Domain organization of the full-length CrfP. *SP*, signal peptide; CHAP, the catalytic module, *SH3b*, bacterial SH3b module. *S*chematic representation of CrfP overall structure. The CHAP and SH3b are colored in blue and yellow, respectively. (B) The protein was purified from pET-28a. The protein in SDS-PAGE gel that stained with Coomassie blue R250. Lane 1 was purified CrfP with HisTrap column and line 2 was crude extract from cells without purified. Line 3, M, mean protein ladder marker. (C) The protein in figure A was analyzed with Western blot. The bands indicate the 36 kD CrfP protein.

**Additional file 5. Phylogenetic analysis of CrfP homologous proteins from *Streptococcus*** **species.** (A) Evolutionary relationship between murein hydrolases from different species. Three branches were formed based on amino acid differences, and the diverse serotype SS belonged to the same group and was far from the other species. A neighbour-joining tree (bootstrap n = 1000; Poisson correction) was constructed based on a ClustalW alignment of the amino acid sequences using MEGA software version 5.0. (B) CrfP protein from different *S.* *suis* strains showed high amino acid sequence similarity. The green background indicates similarity >75%, and the blue background indicates similarity >100%.

**Additional file 6. Biological characteristics analysis between WT and** **ΔcrfP.** (A) PCR results for deletion of *crfP* gene. (B) Microscopy observation of wild type and mutant. The SS was stain with crystal. (C) Growth curve analysis between wild type and mutant. (D) The biofilms were quantified using a multifunctional microplate reader at OD595. We investigated the potential roles of CrfP in *S.* *suis* biological ability. Initially, the growth properties in THB culture were compared between the wild type and mutant, and the growth curve was the same in each phase. Gram staining for microscopy showed that ZY05719 and Δ*crfP* had similar chain lengths, suggesting that there was no correlation between CrfP activity and modulation of *S.* *suis* chain length. In addition, bacterial biofilms are composed of extracellular DNA and glycoproteins, which contribute to host immune defence or antimicrobial resistance. These components of biofilms are products of lytic processes. To evaluate whether CrfP is involved in *S.* *suis* biofilm formation, a crystal violet staining assay was used. However, the OD_595_ data showed no difference between Δ*crfP* and the parental strain, which suggested that CrfP did not mediate biofilm formation in *S.* *suis.*

## Data Availability

All data generated or analyse during this study are included in this published article and its supplementary information files.

## Abbreviations

*S.**suis*: *Streptococcus* *suis*
THB: Todd–Hewitt broth
TCS: Two-component signalling system
CrfP: ComX-related fratricide protein
XIP: ComX-inducing peptide
OD600: Optical density at 600 nm
qRT-PCR: Quantitative real-time polymerase chain reaction
SPF: Specific pathogen-free
PAI: Pathogenicity island
LD50: Median lethal dose

## References

[1] Bonifait L, Veillette M, Letourneau V, Grenier D, Duchaine C (2014). Detection *of**Streptococcus**suis* in bioaerosols of swine confinement buildings. Appl Environ Microbiol.

[2] Feng Y, Zhang H, Wu Z, Wang S, Cao M, Hu D, Wang C (2014). *Streptococcus**suis* infection: an emerging/reemerging challenge of bacterial infectious diseases?. Virulence.

[3] Huang J, Liu X, Chen H, Chen L, Gao X, Pan Z, Wang J, Lu C, Yao H, Wang L, Wu Z (2019). Identification of six novel capsular polysaccharide loci (NCL) from *Streptococcus**suis* multidrug resistant non-typeable strains and the pathogenic characteristic of strains carrying new NCLs. Transbound Emerg Dis.

[4] Pan Z, Ma J, Dong W, Song W, Wang K, Lu C, Yao H (2015). Novel variant serotype of *streptococcus**suis* isolated from piglets with meningitis. Appl Environ Microbiol.

[5] Zheng H, Ji S, Liu Z, Lan R, Huang Y, Bai X, Gottschalk M, Xu J (2015). Eight novel capsular polysaccharide synthesis gene loci identified in nontypeable *Streptococcus**suis* isolates. Appl Environ Microbiol.

[6] Chewapreecha C, Marttinen P, Croucher NJ, Salter SJ, Harris SR, Mather AE, Hanage WP, Goldblatt D, Nosten FH, Turner C, Turner P, Bentley SD, Parkhill J (2014). Comprehensive identification of single nucleotide polymorphisms associated with beta-lactam resistance within pneumococcal mosaic genes. PLoS Genet.

[7] Shanker E, Morrison DA, Talagas A, Nessler S, Federle MJ, Prehna G (2016). Pheromone recognition and selectivity by ComR proteins among *Streptococcus* species. PLoS Pathog.

[8] Claverys JP, Prudhomme M, Martin B (2006). Induction of competence regulons as a general response to stress in gram-positive bacteria. Annu Rev Microbiol.

[9] Hiller NL, Janto B, Hogg JS, Boissy R, Yu S, Powell E, Keefe R, Ehrlich NE, Shen K, Hayes J, Barbadora K, Klimke W, Dernovoy D, Tatusova T, Parkhill J, Bentley SD, Post JC, Ehrlich GD, Hu FZ (2007). Comparative genomic analyses of seventeen *Streptococcus**pneumoniae* strains: insights into the pneumococcal supragenome. J Bacteriol.

[10] Eldholm V, Johnsborg O, Haugen K, Havarstein OHS, LS,  (2009). Fratricide in Streptococcus pneumoniae: contributions and role of the cell wall hydrolases CbpD, LytA and LytC. Microbiology.

[11] Eldholm V, Johnsborg O, Straume D, Ohnstad HS, Berg KH, Hermoso JA, Havarstein LS (2010). Pneumococcal CbpD is a murein hydrolase that requires a dual cell envelope binding specificity to kill target cells during fratricide. Mol Microbiol.

[12] Bateman A, Rawlings ND (2003). The CHAP domain: a large family of amidases including GSP amidase and peptidoglycan hydrolases. Trends Biochem Sci.

[13] Rigden DJ, Jedrzejas MJ, Galperin MY (2003). Amidase domains from bacterial and phage autolysins define a family of gamma-D, L-glutamate-specific amidohydrolases. Trends Biochem Sci.

[14] Biornstad TJ, Ohnstad HS, Havarstein LS (2012). Deletion of the murein hydrolase CbpD reduces transformation efficiency in *Streptococcus**thermophilus*. Microbiology.

[15] Zaccaria E, Wels M, van Baarlen P, Wells JM (2016). Temporal regulation of the transformasome and competence development in Streptococcus suis. Front Microbiol.

[16] Li Q, Fu Y, Ma C, He Y, Yu Y, Du D, Yao H, Lu C, Zhang W (2017). The non-conserved region of MRP is involved in the virulence of *Streptococcus**suis* serotype 2. Virulence.

[17] Kelley LA, Mezulis S, Yates CM, Wass MN, Sternberg MJ (2015). The Phyre2 web portal for protein modeling, prediction and analysis. Nat Protoc.

[18] Kelley LA, Sternberg MJ (2009). Protein structure prediction on the Web: a case study using the Phyre server. Nat Protoc.

[19] Bingle LE, Bailey CM, Pallen MJ (2008). Type VI secretion: a beginner's guide. Curr Opin Microbiol.

[20] Arnold K, Bordoli L, Kopp J, Schwede T (2006). The SWISS-MODEL workspace: a web-based environment for protein structure homology modelling. Bioinformatics.

[21] Kiefer F, Arnold K, Kunzli M, Bordoli L, Schwede T (2009). The SWISS-MODEL Repository and associated resources. Nucleic Acids Res.

[22] Zaccaria E, van Baarlen P, de Greeff A, Morrison DA, Smith H, Wells JM (2014). Control of competence for DNA transformation in Streptococcus suis by genetically transferable pherotypes. PLoS ONE.

[23] Zhu Y, Dong W, Ma J, Zhang Y, Pan Z, Yao H (2019). Utilization of the ComRS system for the rapid markerless deletion of chromosomal genes in Streptococcus suis. Future Microbiol.

[24] Zhong X, Zhang Y, Zhu Y, Dong W, Ma J, Pan Z, Roy S, Lu C, Yao H (2018). The two-component signaling system VraSRss is critical for multidrug resistance and full virulence in Streptococcus suis serotype 2. Infect Immun.

[25] Ma J, Bao Y, Sun M, Dong W, Pan Z, Zhang W, Lu C, Yao H (2015). Two functional type VI secretion systems in avian pathogenic Escherichia coli are involved in different pathogenic pathways. Infect Immun.

[26] Zhang Y, Lu P, Pan Z, Zhu Y, Ma J, Zhong X, Dong W, Lu C, Yao H (2018) Sssp1, a *Streptococcus**suis* fimbriae-like protein transported by SecY2/A2 system contributes to bacterial virulence. Appl Environ Microbiol e01385–18 10.1128/AEM.01385-18 PMC6122003 30030221

[27] Nelson D, Loomis L, Fischetti VA (2001). Prevention and elimination of upper respiratory colonization of mice by group A streptococci by using a bacteriophage lytic enzyme. Proc Natl Acad Sci USA.

[28] Zhang H, Zhang C, Wang H, Yan Y, Sun J (2016) A novel prophage lysin Ply5218 with extended lytic activity and stability against *Streptococcus**suis* infection. FEMS Microbiol Lett 363 10.1093/femsle/fnw186 27481700

[29] Bojarska A, Molska E, Janas K, Skoczynska A, Stefaniuk E, Hryniewicz W, Sadowy E (2016). Streptococcus suis in invasive human infections in Poland: clonality and determinants of virulence and antimicrobial resistance. Eur J Clin Microbiol Infect Dis.

[30] Ma F, Yi L, Yu N, Wang G, Ma Z, Lin H, Fan H (2017). *Streptococcus**suis* serotype 2 biofilms inhibit the formation of neutrophil extracellular traps. Front Cell Infect Microbiol.

[31] Havarstein LS, Martin B, Johnsborg O, Granadel C, Claverys JP (2006). New insights into the pneumococcal fratricide: relationship to clumping and identification of a novel immunity factor. Mol Microbiol.

[32] Luo P, Morrison DA (2003). Transient association of an alternative sigma factor, ComX, with RNA polymerase during the period of competence for genetic transformation in Streptococcus pneumoniae. J Bacteriol.

[33] Johnston C, Martin B, Fichant G, Polard P, Claverys JP (2014). Bacterial transformation: distribution, shared mechanisms and divergent control. Nat Rev Microbiol.

[34] Mashburn-Warren L, Morrison DA, Federle MJ (2010). A novel double-tryptophan peptide pheromone controls competence in Streptococcus spp. via an Rgg regulator. Mol Microbiol.

[35] Heng NC, Tagg JR, Tompkins GR (2007). Competence-dependent bacteriocin production by S*treptococcus**gordonii* DL1 (Challis). J Bacteriol.

[36] Martin B, Quentin Y, Fichant G, Claverys JP (2006). Independent evolution of competence regulatory cascades in streptococci?. Trends Microbiol.

[37] Zhang Y, Zhong X, Lu P, Zhu Y, Dong W, Roy S, Hejair HM, Pan Z, Ma J, Yao H (2019). A novel autolysin AtlASS mediates bacterial cell separation during cell division and contributes to full virulence in Streptococcus suis. Vet Microbiol.

[38] Johnsborg O, Eldholm V, Bjornstad ML, Havarstein LS (2008). A predatory mechanism dramatically increases the efficiency of lateral gene transfer in Streptococcus pneumoniae and related commensal species. Mol Microbiol.

[39] Dubnau D (1999). DNA uptake in bacteria. Annu Rev Microbiol.

[40] Peterson SN, Sung CK, Cline R, Desai BV, Snesrud EC, Luo P, Walling J, Li H, Mintz M, Tsegaye G, Burr PC, Do Y, Ahn S, Gilbert J, Fleischmann RD, Morrison DA (2004). Identification of competence pheromone responsive genes in Streptococcus pneumoniae by use of DNA microarrays. Mol Microbiol.

[41] Berg KH, Biornstad TJ, Johnsborg O, Havarstein LS (2012). Properties and biological role of streptococcal fratricins. Appl Environ Microbiol.

[42] Berg KH, Ohnstad HS, Havarstein LS (2012). LytF, a novel competence-regulated murein hydrolase in the genus Streptococcus. J Bacteriol.

[43] Bublitz M, Polle L, Holland C, Heinz DW, Nimtz M, Schubert WD (2009). Structural basis for autoinhibition and activation of Auto, a virulence-associated peptidoglycan hydrolase of Listeria monocytogenes. Mol Microbiol.

[44] Jedrzejas MJ (2001). Pneumococcal virulence factors: structure and function. Microbiol Mol Biol Rev.

[45] Lenz LL, Mohammadi S, Geissler A, Portnoy DA (2003). SecA2-dependent secretion of autolytic enzymes promotes Listeria monocytogenes pathogenesis. Proc Natl Acad Sci USA.

[46] Ramos-Sevillano E, Urzainqui A, Campuzano S, Moscoso M, Gonzalez-Camacho F, Domenech M, Rodriguez de Cordoba S, Sanchez-Madrid F, Brown JS, Garcia E, Yuste J (2015). Pleiotropic effects of cell wall amidase LytA on *Streptococcus**pneumoniae* sensitivity to the host immune response. Infect Immun.

