# Supplementary material for: CrfP, a fratricide protein, contributes to natural transformation in Streptococcussuis
Source: Vet Res. 2021 Mar 24;52:50. doi: 10.1186/s13567-021-00917-x (PMC7992943; doi:10.1186/s13567-021-00917-x)
Supplement: Supplementary file 1 — Additional file 1. Characteristics of the bacterial strains and plasmids used in this study. [file 13567_2021_917_MOESM1_ESM.docx]

**Additional file 1 Characteristics of the bacterial strains and plasmids used in this study**

| Strains or plasmids | Characteristic | Sources |
| --- | --- | --- |
| **Plasmids** |  |  |
| pSET-2 | *S. suis* complemention plasmid, spc^R^ | Stored in Lab |
| pET-28a | Expression vector, Kan^R^ | Stored in Lab |
| pET-28a-CrfP | pET-28a carrying *crfp* coding region | This study |
| pET-28a-CHAP | pET-28a carrying *chap* coding region | This study |
| pET-28a-SH3-RFP | pET-28a carrying *sh3* and *rfp* coding region | This study |
| pSET-2::*spa* | The vector with 4*flag used express protein in *S.suis* | Stored in Lab |
| **Strains** |  |  |
| XL10 | For cloning the recombinant plasmids, Cm^R^ | Vazyme |
| DH5α | For cloning the recombinant plasmids | Vazyme |
| BL21 | Host for expressing the recombinant proteins | Vazyme |
| MC1061 | For cloning the recombinant plasmids | Stored in Lab |
| ZY05719 | Isolated from a diseased pig in China; Virulent SS2 strain | Stored in Lab |
| Δ*crfR* | Isogenic *crf* mutant of strain ZY05719 | This study |
| P1/7 | Isolated from a diseased pig in UK; Virulent SS2 strain, ST1 | Stored in Lab |
| HA0609 | Isolated from Haian, SS2, ST28 | Stored in Lab |
| SC070731 | Isolated from Sichuan, SS2, ST7 | Stored in Lab |
| T15 | Purchase from ATCC, SS2, ST28 | Stored in Lab |
| 128-1-2 | unknown, SS3, ST28 | Stored in Lab |
| 129-3-3 | unknown, SS3, ST28 | Stored in Lab |
| HN105 | Isolated from Henan, SS5, ST498 | Stored in Lab |
| Sdly140101 | Isolated from Shandong, SS5, ST498 | Stored in Lab |
| SD16062008 | Isolated from Shandong, SS9, unknown | Stored in Lab |
| GZ0565 | Isolated from Guangzhou, SS9, ST243 | Stored in Lab |
| CZ130302 | Isolated from Changzhou , SS Chz, ST383 | Stored in Lab |
| HN136 | Isolated from Henan , SS Chz, ST264 | Stored in Lab |
| AH681 | Isolated from Anhui , SS Chz, ST475 | Stored in Lab |
| A909 | Purchase from ATCC, *S.agalactiae* | Stored in Lab |
| GD201008-001 | Isolated from Changdong*, S.agalactiae* | Stored in Lab |
| 201706NMGCOW | Isolated from Neimenggu, *S.agalactiae* | Stored in Lab |
| 201706NMGCOWB1 | Isolated from Neimenggu, *S.agalactiae* | Stored in Lab |
| 201706NMGCOWB2 | Isolated from Neimenggu, *S.agalactiae* | Stored in Lab |
| ATCC35246 | Purchase from ATCC, *S.equi* | Stored in Lab |
| BV2 | Unknown, *S.aureus* | Stored in Lab |
| BV33 | Unknown, *S.aureus* | Stored in Lab |
| 201707YRXM | Unknown, *S.uberis* | Stored in Lab |
| 201705HHHTCOW | Unknown, *S.uberis* | Stored in Lab |
