# Supplementary material for: CrfP, a fratricide protein, contributes to natural transformation in Streptococcussuis
Source: Vet Res. 2021 Mar 24;52:50. doi: 10.1186/s13567-021-00917-x (PMC7992943; doi:10.1186/s13567-021-00917-x)
Supplement: Supplementary file 2 — Additional file 2. Locus tags of competence-related murein hydrolases and pili from different Streptococcus species. [file 13567_2021_917_MOESM2_ESM.docx]

**Additional file 2 Locus tags of competence-related murein hydrolases and pili from different *Streptococcus* species**

| **Strains** | **Locus tag** |
| --- | --- |
|  | Murein **hydrolases** |
| *S. pneumoniae* R6 | spr2006 |
| *S. mitis* NCTC 12261 | SM12261_0760 |
| *S. oralis* Uo5 | SOR_1962 |
| *S. uberis* 0140J | SUB0048 |
| *S. pyogenes* M1 GAS | SPy_0031 |
| *S. equi* subsp. ATCC 35246 | SeseC_00035 |
| *S. equi* subsp. 4047 | SEQ_0031 |
| *S. thermophiles* LMG | stu0039 |
| *S. salivarius* SK126 | STRSA0001_1425 |
|  | **Pilus** |
| *S. suis* ZY05719 ComYA | ZY05719_00770 |
| *S. suis* ZY05719 ComYB | ZY05719_00775 |
| *S. suis* ZY05719 ComYC | ZY05719_00780 |
| *S. suis* ZY05719 ComYD | ZY05719_00785 |
| *S. suis* ZY05719 ComYE | ZY05719_00790 |
| *S. suis* ZY05719 ComYF | ZY05719_00795 |
| *S. suis* ZY05719 ComYG | ZY05719_00800 |
| *S. suis* ZY05719 ComYH | ZY05719_00805 |
