# Supplementary material for: CrfP, a fratricide protein, contributes to natural transformation in Streptococcussuis
Source: Vet Res. 2021 Mar 24;52:50. doi: 10.1186/s13567-021-00917-x (PMC7992943; doi:10.1186/s13567-021-00917-x)
Supplement: Supplementary file 3 — Additional file 3. Primers used in this study. [file 13567_2021_917_MOESM3_ESM.docx]

**Additional file 3 Primers used in this study.**

| Primer | Sequence(5’-3’) | Function or genes |
| --- | --- | --- |
| CrfP-F | AGAAGGAGATATACCATGGGGGCAAGAGGAGATGACTATCCC | CrfP |
| CrfP-R | GTGGTGGTGGTGCTCGAGTAAATATTGTATTTTTTGTGATTCG |  |
| CHAP-F | AGAAGGAGATATACCATGGGGGCAAGAGGAGATGACTATCCC | CHAP |
| CHAP-R | GTGGTGGTGGTGCTCGAGGGTTGTTCCCGCTCCGAGGTT |  |
| SH3-1 | AGAAGGAGATATACCATGGGGAACTCTCAACCCGCCTTAGCC | SH3 |
| SH3-2 | cgcctccagaacctcctccaccTAAATATTGTATTTTTTGTGATTCG |  |
| SH3-3 | gaggttctggaggcggtggaagtggtggcggaggtagcATGGCCTCCTCCGAGAACGTC | GFP |
| SH3-4 | GTGGTGGTGGTGCTCGAGCTACAGGAACAGGTGGTGGCG |  |
| qcrf-F | AGAGGAGATGACTATCCCTACAC | qRT-PCR for *crfP* |
| qcrf-R | GTGCTAAGTCGAAAGGCTACA |  |
| crfPspa-F | CCGGAATTCATCAAAGCCTTCGGCCATCAG | *crfP* gene insert into  pSET-2:spa |
| crfPspa-R | CGCGGATCCGGAGATGGCGATGTAGCGTCGAG |  |
| q00195-F | AGCAACAAGTAGCCTCAGTATC | qRT-PCR  ZY05719_00195 |
| q00195-R | TGTGCTTACCGTTGGTGTAG |  |
| q04700-F | CAAGGATGGAACGGGTCTAAA | qRT-PCR for  ZY05719_04700 |
| q04700-R | GGTGGTCTGAACCTGGATTAC |  |
| q09810-F | TGTCAGAGGTTGCTTTGGAATA | qRT-PCR for  ZY05719_09810 |
| q09810-R | TGCCCAATCTCCACCATTAC |  |
| parC-F | TGGAGATGCACGGAAACAA | housekeeping |
| parC-R | CTCGATGTCAGCCAAGAGATAG | gene for *S. suis* |
| crfP-1 | GTTGATTGTTGCCTACAAACT | Delete *crfP* gene in  ZY05719 |
| crfP-2 | TTCAGCATTATCCTATTTCTCCTTGTGTAGTAGA |  |
| crfP-3 | ACCCATCGAATTATCACAAAAAATACAATATTTA |  |
| crfP-4 | ACATAACCATCCTAATAATGT |  |
| crfP-5 | TTCTACAACGATAAGATGGAC |  |
| crfP-6 | GAACTCCTCTAAGCAGCTAAC |  |
| JCcrfP-F | AATGCAAGAGGAGATGACTAT | Detect the *crfP* gene |
| JCcrfP-R | ATAACTTTATCATAGCGAACG |  |
| *sacB-cm*-F | GGATAATGCTGAAAACTCCTT | *sacB-cm* cassette |
| *sacB-cm*-R | TAATTCGATGGGTTCCGAGG |  |
| comR -1 | CGCGGATCCTGCGGACAAGTACGGTCTGGA | Left flanking regions  of *comR* |
| comR -2 | AAACCACACCTCCAAATATAC |  |
| comR -3 | AGGTGTGGTTTAGTGACATTTTTGTCCTGTTG | Right flanking regions  of *comR* |
| comR -4 | AAAACTGCAGAGCTTGATACCACCTGACTGA |  |
| JCcomR-F | AACGATAAGGAATTTGGACAG | PCR for *comR* gene |
| JCcomRS-R | TTATTCTTCAACCCATGTTCC |  |
| sacB-spc-F | GGATAATGCTGAAAACTCCTT | *sacB-spc* casette |
| sacB-spc-R | AATCTGATTACCAATTAGAATGA |  |
| comX-1 | TAACGATGAACGTGAACAAACC | Left flanking regions  of *comX* |
| comX-2 | TTCAGCATTATCCTACTTAATCTCCTTTTGTTGAT |  |
| comX-3 | ACCCATCGAATTAAGCTAGTTACTAACATCACGTT | Right flanking regions  of *comX* |
| comX-4 | GTCAATTCCTTTGAGTTTCAAC |  |
| comX-5 | ATTAGTAGCACAACAAACACCA | Fusion primers |
| comX-6 | TTACAACAGAGCTTTACGATCC |  |
| JCcomX-F | TTTACATTAAACTATGGGATC | Detection primers for  *comX* |
| JCcomX-R | AATTAATTTTTCGTATTTATC |  |
| qcomR-F | AACTCTCTGTGCGCCAATTA | qRT-PCR for *comR* |
| qcomR-R | GCTCGTAAAGTCCCATACCTAAA |  |
| qcomX-F | GACCGTATGCCACATGAAGAT | qRT-PCR for *comX* |
| qcomX-R | CTCAAGGCACCTCTCAACATTA |  |
| qcomYA-F | GCCATCATCAGCCATTTCAAG | qRT-PCR for *comYA* |
| qcomYA-R | AAATCTCTCCGCTCCCATAATC |  |
| qcomYB-F | GGGTAGCGAGTTGACAGTTTAT | qRT-PCR for *comYB* |
| qcomYB-R | AAGACCAGCGGTTGGATTAG |  |
| qssbB-F | TACTAGAAAGCCGTGCTCAAC | qRT-PCR for *ssbB* |
| qssbB-R | AAACGGTAATTCCTCCTCTTCC |  |
