# Supplementary figures and images for: CrfP, a fratricide protein, contributes to natural transformation in Streptococcussuis
Source: Vet Res. 2021 Mar 24;52:50. doi: 10.1186/s13567-021-00917-x (PMC7992943; doi:10.1186/s13567-021-00917-x)

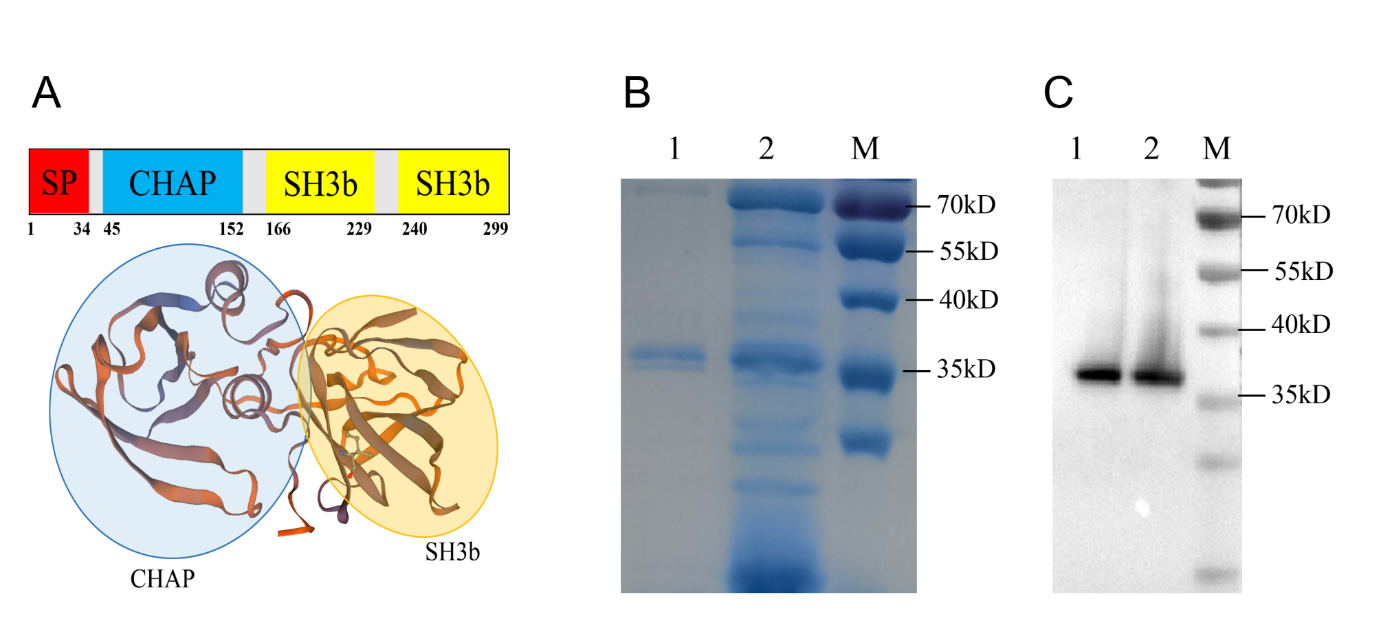

Supplement: Supplementary file 4 — Additional file 4. Structure and Western blot analysis of His tagged CrfP protein. (A) Domain organization of the full-length CrfP. SP, signal peptide; CHAP, the catalytic module, SH3b, bacterial SH3b module. Schematic representation of CrfP overall structure. The CHAP and SH3b are colored in blue and yellow, respectively. (B) The protein was purified from pET-28a. The protein in SDS-PAGE gel that stained with Coomassie blue R250. Lane 1 was purified CrfP with HisTrap column and line 2 was crude extract from cells without purified. Line 3, M, mean protein ladder marker. (C) The protein in figure A was analyzed with Western blot. The bands indicate the 36 kD CrfP protein. [file 13567_2021_917_MOESM4_ESM.docx]

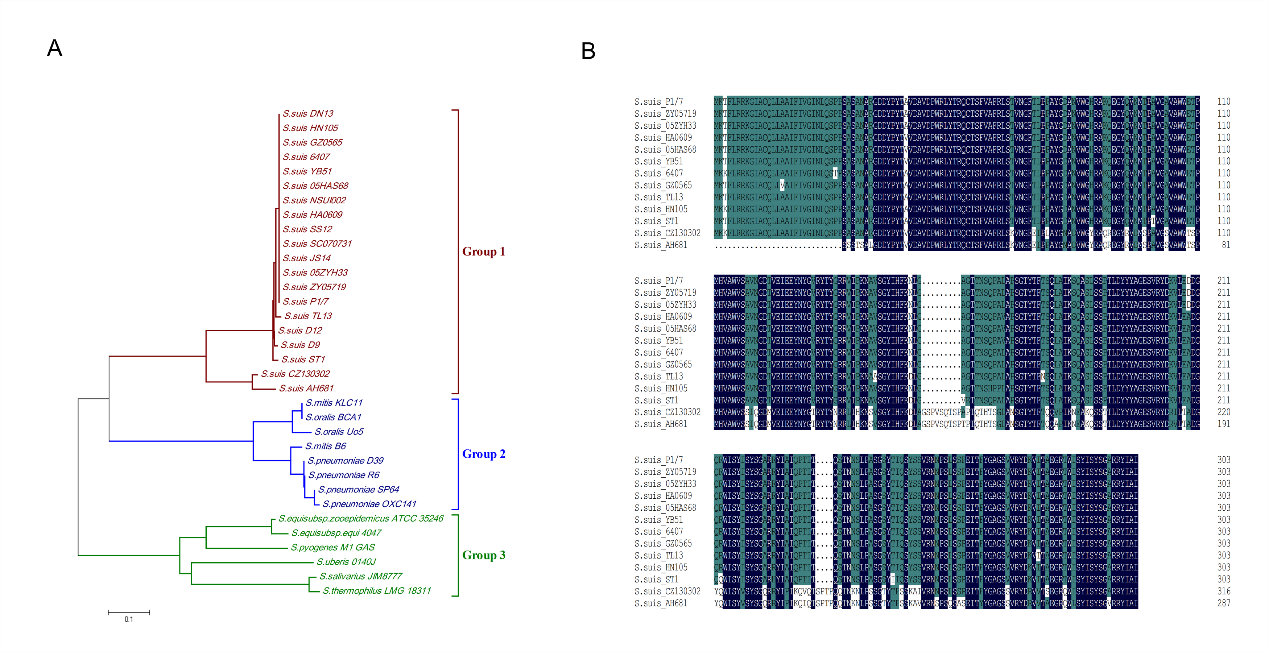

Supplement: Supplementary file 5 — Additional file 5. Phylogenetic analysis of CrfP homologous proteins from Streptococcus species. (A) Evolutionary relationship between murein hydrolases from different species. Three branches were formed based on amino acid differences, and the diverse serotype SS belonged to the same group and was far from the other species. A neighbour-joining tree (bootstrap n = 1000; Poisson correction) was constructed based on a ClustalW alignment of the amino acid sequences using MEGA software version 5.0. (B) CrfP protein from different S. suis strains showed high amino acid sequence similarity. The green background indicates similarity >75%, and the blue background indicates similarity >100%. [file 13567_2021_917_MOESM5_ESM.docx]

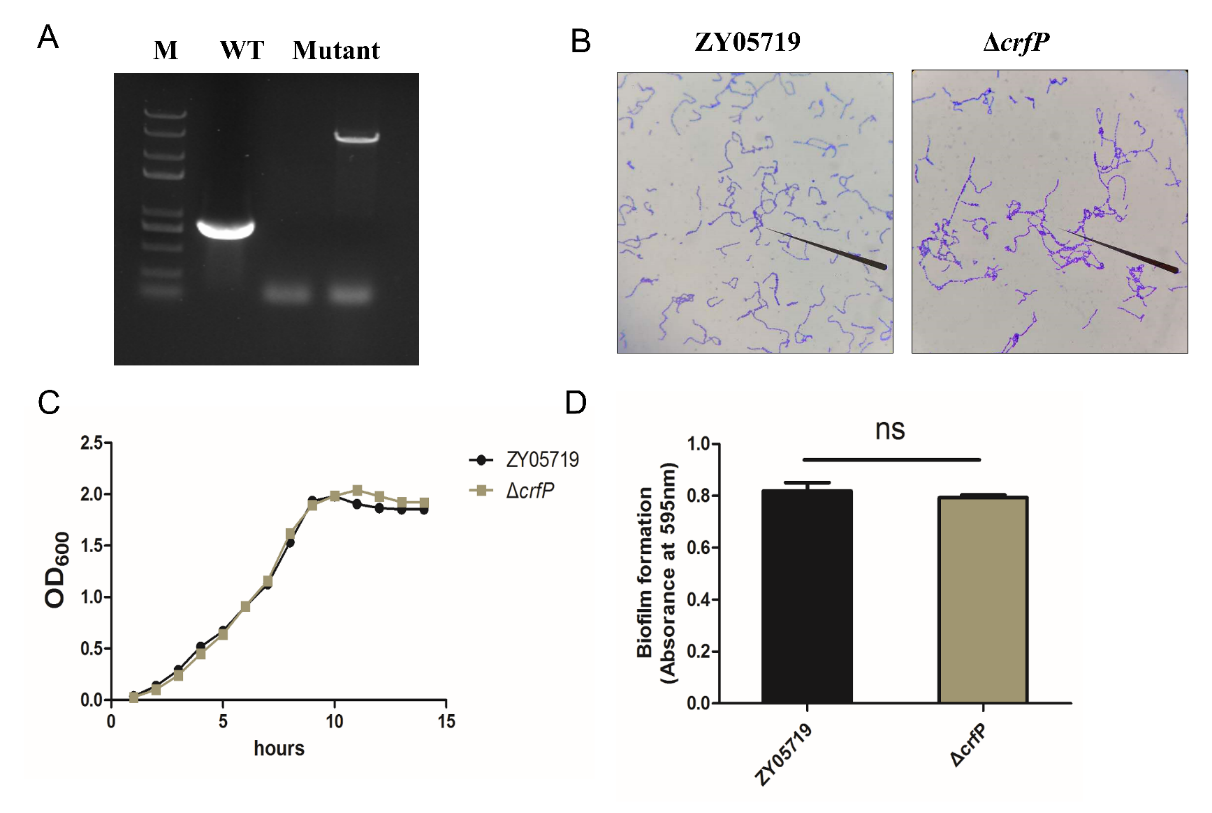

Supplement: Supplementary file 6 — Additional file 6. Biological characteristics analysis between WT and ΔcrfP. (A) PCR results for deletion of crfP gene. (B) Microscopy observation of wild type and mutant. The SS was stain with crystal. (C) Growth curve analysis between wild type and mutant. (D) The biofilms were quantified using a multifunctional microplate reader at OD595. We investigated the potential roles of CrfP in S. suis biological ability. Initially, the growth properties in THB culture were compared between the wild type and mutant, and the growth curve was the same in each phase. Gram staining for microscopy showed that ZY05719 and ΔcrfP had similar chain lengths, suggesting that there was no correlation between CrfP activity and modulation of S. suis chain length. In addition, bacterial biofilms are composed of extracellular DNA and glycoproteins, which contribute to host immune defence or antimicrobial resistance. These components of biofilms are products of lytic processes. To evaluate whether CrfP is involved in S. suis biofilm formation, a crystal violet staining assay was used. However, the OD595 data showed no difference between ΔcrfP and the parental strain, which suggested that CrfP did not mediate biofilm formation in S. suis. [file 13567_2021_917_MOESM6_ESM.docx]
